# Editorial: Drug-ability strategies for potential antimycobacterial candidate: opportunities and challenges

**DOI:** 10.3389/fphar.2023.1294912

**Published:** 2023-11-14

**Authors:** Shasank Sekhar Swain, Sunday Oyedemi, Sudhir Kumar Paidesetty, Soumitra Mohanty, Tahziba Hussain

**Affiliations:** ^1^ Division of Microbiology and NCDs, Regional Medical Research Centre, Bhubaneswar, Odisha, India; ^2^ Department of Pharmacology, School of Science and Technology, Nottingham Trent University, Nottingham, United Kingdom; ^3^ Department of Medicinal Chemistry, School of Pharmaceutical Sciences, Siksha “O” Anusandhan University, Bhubaneswar, Odisha, India; ^4^ Division of Clinical Microbiology, Karolinska University Hospital, Stockholm, Sweden

**Keywords:** anti-TB drug development, drug development strategies against tuberculosis, natural product-based drug discovery, bioinformatics tools, medicinal chemistry and drug synthesis

Tuberculosis (TB) caused by *Mycobacterium tuberculosis* (Mtb) is an infectious killer with a higher prevalence rate (10.6 million) and mortality rate (1.6 million) across the globe as per the WHO-Global Tuberculosis Report-2022 (Swain et al., 2022; WHO, 2022). After continuous scientific research efforts and socio-political awareness campaigns, we are unable to control emerging drug-resistant strains of Mtb. In the last two decades, only three anti-TB drugs (bedaquiline, delanamid, and pretomanid) have been approved by the FDA, which creates havoc in anti-TB therapy as well in primary healthcare system. In addition, long-term anti-TB therapy has shown several common (gastrointestinal intolerance, headache, diarrhoea, etc.) to severe (hepato- and neurotoxic) side effects. Therefore, several ingenious attempts have been made to improve early diagnosis and locate or design potential anti-TB candidates (Acharya et al., 2020; Swain et al., 2020; Dong et al., 2022; Swain and Hussain, 2022). Four articles have been published in the first edition of the Research Topic. As the ultimate goal is to control Mtb, all have used different modalities and expertise to accelerate anti-TB drug development modules, which are introduced here.

Tanner et al. developed three newer versions of the existing anticoccidial drug decoquinate (DQ) through an ingenious medicinal chemistry protocol. DQ is a potent quinolone nucleus-bearing antiparasite medication used to prevent coccidian infections in poultry feed for over 50 years and reported as having no activity against Mtb, with poor drug-likeness and low water solubility profiles. The proposed derivatives (RMB041, RMB043, and RMB073) bearing an alkyl group at N-1 with amide groups at C-3 substitutions exhibited potential anti-TB activity with 90% minimum inhibitory concentrations (MIC_90_), 1.61, 4.18, and 1.88 µM against the Mtb H37Rv strain. Further, DQ-derivatives showed higher selectivity indices (SI, 10–25) against Mtb based on *in vitro* cytotoxicity (IC_50_ = 20, 80, and 33.9 µM) against Chinese hamster ovarian cell lines with improved drug-likeness, kinetic solubility, bioavailability, and PK/toxicity profiles than native DQ. Therefore, the above *in vitro* and *in vivo* investigations revealed that proposed DQ derivatives could be used as an alternative anti-TB candidate after some pharmacological investigation.

Another interesting review article by Naidu et al. introduced the prominence and benefits of bioinformatics and machine learning tools in tuberculosis research. Systematically, the review has been divided into four parts: (i) introduction of available systems and computational tools for biomarker and therapeutic target identification; (ii) computational approaches in drug discovery to screen potential drug-able compounds; (iii) computational platforms for tuberculosis vaccine development; and (iv) future perspectives along with how to overcome the apparent bias and errors during analyses. Overall, virtual screening, molecular docking, pharmacophore modelling, molecular dynamic simulation, toxicity, and drug-ability prediction through various robust, user-friendly, and widely used tools or platforms can accelerate anti-TB drug discovery within a limited resource and time frame.

Drumond et al. demonstrated an adjuvant antibacterial therapy, i.e., cell-free spent medium (CFSM) derived from four lactic acid and probiotic bacteria (*Lactiplantibacillus plantarum*, *Lactobacillus acidophilus*, *Lactobacillus johnsonii*, and *Lactobacillus delbrueckii*), which displayed potential antibacterial and anti-biofilm potency against two distinct strains of *Pseudomonas aeruginosa* (9027™ and 27853™). Using conventional antibacterial testing methodologies such as zone of inhibition (size in mm), minimum inhibitory concentration (MIC), and minimum bactericidal concentration (MBC) in µL against planktonic conditions, along with anti-biofilm potency in three stages (pre-coated, co-incubated, and preformed with crystal violet and MTT assay), it has been shown that CFSM have moderate potency around 54% inhibition at higher concentrations of 300 µL. Overall, the hypothesised adjuvant therapy is an alternative option to reduce the burden of hospital infections and could be used against Mtb.

Another study by Aro et al. investigated the antimycobacterial (against *M. tuberculosis* H37Rv, *M. tuberculosis* ATCC 25177, and *M. bovis* ATCC 27299 strains), immunomodulatory (expression of six cytokines, IL-2, IL-4, IL-5, IL-10, IFN-γ, and TNF-α in the LPS-activated U937 cell line), and apoptosis (annexin V/PI assay) of bio-assay-guided acetone-derived leaf crude extracts of six Rubiaceae species. For scientific validation and utilization of immune-modulating traditional herbal regimens to control immune-related diseases like TB, crude extracts showed potency within a range of 39–312 μg/mL, where *Cremaspora triflora* and *Cephalanthus natalensis* were more active at MIC = 39 μg/mL against Mtb. Among all extracts, *Psychotria zombamontana* and *Psychotria capensis* had significant IC_50_ values of 4.32 and 5.8 μg/mL against 5-lipoxygenase (LOX) and all extracts showed apoptosis in a time- and dose-dependent manner. Overall, this preliminary investigation encourages scientific investigation of enriched traditional herbal extracts as cost-effective alternative therapeutics against MTB, which will help reduce antibiotic consumption and the shortage of anti-TB drugs.

In conclusion, exploring traditional knowledge, structural modification of existing drugs and natural products, hybrid drug development, functionalized nano-formulation, etc., are some ideal approaches that have been used to develop or locate potentially less toxic anti-TB drug regimens (Swain et al., 2020; Baranyai et al., 2021; Dartois and Rubin, 2022; Swain and Hussain, 2022). As a result, several cephalosporins, tetracyclines, and oxazolidinone derivatives are in clinical trials, and several phytoconstituents are also identified as having anti-TB activity with MIC values <1 µM. However, the anti-TB drug approval rate is in decline mode as very few candidates pass the clinical trials as there are lots of drug parameters that need to be satisfied to be a drug-able candidate or regimen (Aleksandrov and Myllykallio, 2019; Swain and Hussain, 2022). Therefore, along with pioneering combinatorial chemistry and advanced instrumentation, computational tools help to accelerate the modern anti-TB drug discovery process by predicting the potency, toxicity, pharmacokinetics, and overall drug-ability profiles at the primary stage (Aleksandrov and Myllykallio, 2019; Swain and Hussain, 2022). Therefore, bioinformatics tools are widely used in the academic as well as pharmaceutical industries to locate drug-able candidates and further proceed with expensive experiments with a higher chance of clinical success.
